# Ongoing hepatitis A among men who have sex with men (MSM) linked to outbreaks in Europe in Tel Aviv area, Israel, December 2016 – June 2017

**DOI:** 10.2807/1560-7917.ES.2017.22.29.30575

**Published:** 2017-07-20

**Authors:** Yael Gozlan, Itay Bar-Or, Aviya Rakovsky, Michal Savion, Ziva Amitai, Rivka Sheffer, Noa Ceder, Emilia Anis, Itamar Grotto, Ella Mendelson, Orna Mor

**Affiliations:** 1 Central Virology Laboratory, Ministry of Health, Sheba Medical Center, Ramat Gan;; 2 Tel-Aviv District Health Office, Ministry of Health, Tel Aviv, Israel;; 3 Public Health Services, Ministry of Health, Jerusalem, Israel;; 4 Hebrew University Hadassah Braun School of Public Health and Community Medicine, Jerusalem; 5 Faculty of Health Sciences, Ben Gurion University of the Negev, Beer Sheva, Israel;; 6 School of Public Health, Tel Aviv University, Tel Aviv

**Keywords:** hepatitis A, hepatitis A virus, sewage, viral hepatitis, viral infections, laboratory

## Abstract

Between December 2016 and June 2017, 19 Hepatitis A virus (HAV)-positive cases, 17 of which were among men who have sex with men (MSM) were identified in the Tel Aviv area. Seven of the 15 sewage samples collected between January and June 2017 were also HAV-positive. All sequences clustered with two of the three strains identified in the current European HAV outbreak. We demonstrate that despite an efficient vaccination programme, HAV can still be transmitted to an unvaccinated high-risk population.

An ongoing hepatitis A outbreak involving 15 European countries with the first case identified in June 2016 in the United Kingdom (UK) [1], was recently reported [2]. By June 2017, 1,173 hepatitis A virus (HAV) infections mainly among men who have sex with men (MSM) were notified. In December 2016, when the first hepatitis A cases in Tel Aviv district were diagnosed, the local public health authority was notified and an ongoing virological, epidemiological and environmental surveillance has commenced. Here we report the current findings of 1 June 2017.

## Identification of hepatitis A virus 1a in clinical and environmental samples

**Figure 1:**
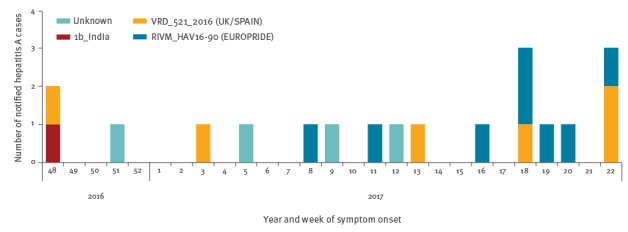
Epidemic curve of hepatitis A cases by risk group, week of onset of illness, and viral strain, December 2016–June 2017, Israel (n = 19)

Hepatitis A is a notifiable disease in Israel and only 17 sporadic cases were reported between March 2013 and December 2016 in the Tel Aviv district, when the first case of the current hepatitis A outbreak was identified in a hospitalised man. A case was defined as a report of an individual who had been in the Tel Aviv health district (covering the Tel Aviv metropolitan region) with a clinical presentation of abrupt onset of hepatitis (fatigue, nausea, anorexia, abnormal liver function tests) and laboratory confirmed anti-HAV (immunoglobulin M, IgM positivity) serology. By June 2017, 19 cases of HAV infection confirmed by serology were notified to the Tel Aviv district health office (Figure 1). Seventeen cases were men, aged 22 to 41 years, who self-identified as MSM. One was a woman with a travel history to India during the incubation period. Of the 15 MSM, three had travelled to Europe, and one to the United States (US) in the two months before symptom onset. Fifteen infections were also confirmed by RT-PCR and sequencing: eight MSM were infected by the HAV 1a_ RIVM_HAV16–90 (EUROPRIDE) and six by the HAV 1a_ VRD_521_2016 (UK/SPAIN) strain; the woman was infected with an HAV 1b_India strain. The Table shows the characteristics of the patients, none of whom were vaccinated.

UK: United Kingdom.

**Table:** Characteristics of notified hepatitis A cases, Tel-Aviv, December 2016-June 2017 (n = 19)

	Other	MSM cluster^a^
Cases	2	17
Median Age (range)	48.5 (35–62)	31 (22–41)
Male sex	1	17
Hospitalised	2	17
Travel in 2 months before symptom onset (detination)	1 (India)	4 (3 Europe, 1 United States)
HIV infection	0	1
Sexual contacts in non-household venues	0	3^b^
Hepatitis A vaccination	0	0
Hepatitis A strain (n = 15)		
1a_ RIVM_HAV16–90 (EUROPRIDE)	NA	8
1a_ VRD_521_2016 (UK/SPAIN)	NA	6
1b_India	1	NA

MSM: men who have sex with men; NA: not applicable; UK: United Kingdom.

^a^Self-identified as MSM.

^b^All were infected with RIVMHAV1690 (EUROPRIDE).

To support the outbreak investigation, the presence of HAV sequences was assessed in sewage samples (n = 23) collected between August 2016 and June 2017 as part of the routine polio surveillance programme [3] from three facilities located in the Tel Aviv area.

All sewage samples collected between August and the end of December 2016 were HAV- negative. Seven of the fifteen samples (3/7 from the Shafdan, the sewage treatment plant of Tel Aviv, and 4/4 samples from a southern Tel Aviv pipeline), collected between January and June 2017, were found to be HAV-positive.

Phylogenetic analysis of clinical and sewage HAV-positive samples showed that all sequences from the current outbreak among MSM in Israel and the seven positive sewage samples, clustered with either RIVM_HAV16–90 (EUROPRIDE) or VRD_521_2016 (UK/SPAIN) isolates identified in the 2016–2017 European HAV outbreak [1,4,5].

Three MSM were infected with the VRD_521_2016 (UK/SPAIN) strain and epidemiologically-linked to the same non-household venue. Moreover, sequencing results were available for three of four MSM who reported travelling in the 2 months before symptoms onset. One of them, identified with the VRD_521_2016 (UK/SPAIN) strain in December 2016 (week 52), was most likely infected in Berlin. Another adult MSM, harbouring the RIVM_HAV16–90 (EUROPRIDE) strain and diagnosed in May 2017 (week 18), reported a stay in Poland during the incubation period. The third MSM, diagnosed in late May (week 22), presented with acute hepatitis two weeks after returning from New York and carried the VRD_521_2016 (UK/SPAIN) strain. All other cases had not travelled abroad and were infected in Israel.

**Figure 2:**
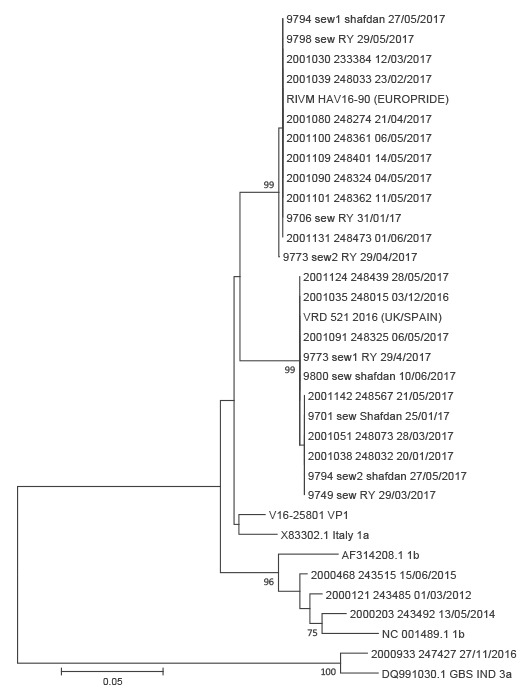
Phylogenetic analysis of virus strains from hepatitis A cases and sewage isolates, 2012–2017, Israel

HAV isolates from sewage also clustered with both HAV strains implicated in the ongoing European outbreak. In two of the sewage samples (9,794 and 9,773), both strains could be identified together. HAV sequences from past outbreaks in Tel Aviv formed a separate cluster with HAV 1b sequences (Figure 1, 2).

Neighbour-joining tree of sequences of 427 nt of the VP1/P2A region of hepatitis A virus strains. The tree was constructed in MEGA 6.0. Strains are identified by sample number, patient number (or sewage facility name) and date of infection (or date of sewage sampling). For comparison, hepatitis A virus strains detected in recent years in Israel and several reference strains (denoted by GeneBank number) were included. Numbers at the nodes indicate bootstrap values.

## Laboratory investigation

Serum or plasma samples from acute hepatitis A cases positive for anti-HAV IgM were transferred to the national centre of HIV and viral hepatitis in the central virology laboratory of the Ministry of Health. Total nucleic acids were purified from 400 µL of plasma (or from 500 µL of filtered and concentrated sewage samples) and real-time PCR for HAV detection was performed as described previously [6]. PCR and sequencing primers of a 460-nt fragment located within the VP1/P2A region were chosen according to the HAVNET unified typing protocol [7]. Sequencing was performed on an ABI 3500 Genetic Analyzer (Applied Biosystems, Foster City, California, US) using an ABI PRISM BigDye Terminator Cycle Sequencing kit (Applied Biosystems). Raw sequence data was analysed corrected and trimmed to generate a 427-nt consensus sequence using Sequencher 5.4 (GeneCodes, Ann Arbor, Michigan, US). The resulting HAV sequences were aligned with the three distinct HAV 1a strains representing the current HAV MSM outbreak in Europe and with sequences from previously identified HAV infections in Israel. Phylogenetic analysis was conducted using a neighbour-joining algorithm in MEGA, version 6 [8], with 1,000 replicates for bootstrap testing.

## Discussion

Hepatitis A virus infection causes a substantial number of viral hepatitis cases worldwide. It is an acute self-limiting illness, associated with fever, malaise, nausea, anorexia and jaundice, mainly transmitted via the faecal-oral route [9]. A two-dose universal toddler's vaccination programme at 1.5 and 2 years of age, was initiated in Israel in 1999 and has led to an over 90% decline in incidence of the disease [10]. However, the programme cannot prevent HAV infection in high risk groups like MSM born in Israel before 1999.

Between March 2012 and March 2013, there was an outbreak in the Tel Aviv district with 75 cases of acute hepatitis, of which 73% were in non-vaccinated young men. HAV 1b was the predominant subtype identified in clinical as well as in sewage samples collected during the outbreak [6]. In the ongoing HAV outbreak occurring in the Tel Aviv district, in non-vaccinated, MSM in the age-group of 20-45 years, two of the three strains currently circulating in MSM in Europe were identified. They were detected in clinical samples from patients and in samples collected from sewage facilities located in the Tel Aviv area. Interestingly, none of the sewage samples collected in the 5 months before the first HAV case was identified, were HAV-positive. We therefore assume that these strains did not circulate in Tel Aviv before December 2016. Moreover, a different HAV subtype, HAV 1b, dominated in the 2012–13 outbreak that affected mainly non-vaccinated injecting drug users and not MSM. The identification of individuals that reported being infected while abroad also supports the conclusion that the current outbreak was imported to Israel by travelling MSM.

Anti-HAV vaccine is routinely recommended by the Israeli Ministry of Health to various risk groups, including MSM [11]. Following the identification of the first hepatitis A cases in MSM in Tel Aviv, we communicated about the outbreak through social media and offered free of charge vaccinations at all public health offices and sex clinics in Israel. Leaflets explaining HAV transmission and calling for anti-HAV vaccination were distributed at the venue where three infections occurred. Controlling the outbreak and protecting those who are not immune is complicated by undernotification as well as by possible asymptomatic HAV infection leading to continuous virus circulation. The abundance of HAV isolates in the environmental samples indicates that not all HAV cases have been identified.

In the past, molecular and phylogenetic analysis of HAV-positive cases in Israel was only done in specific cases. Following the 2012-13 outbreak, the need for a national diagnostic laboratory to support public health decisions on hepatitis A was emphasised. In June 2017, the viral hepatitis reference laboratory in the central virology laboratory of the Ministry of Health was formally established and an official requirement to send IgM-positive HAV samples for molecular confirmation put in place. Thus future outbreaks are expected to be better assessed. Our findings in the MSM population together with the current reports from Europe and recently also in North America [12] call for introduction of new methodologies aiming to increase vaccination coverage specifically within this risk group.

## References

[1] Beebeejaun K Degala S Balogun K Simms I Woodhall SC Heinsbroek E Outbreak of hepatitis A associated with men who have sex with men (MSM), England, July 2016 to January 2017. Euro Surveill. 2017;22(5):30454. 10.2807/1560-7917.ES.2017.22.5.30454 28183392 PMC5388117

[2] European Centre for Disease Prevention and Control (ECDC). Communicable Disease Threats Report (CTDR): Week 23, 4-10 June 2017. ECDC. Jun 2017. Available from https://ecdc.europa.eu/sites/portal/files/documents/Communicable-disease-threats-report-9-june-2017.pdf

[3] Manor Y Shulman LM Kaliner E Hindiyeh M Ram D Sofer D Intensified environmental surveillance supporting the response to wild poliovirus type 1 silent circulation in Israel, 2013. Euro Surveill. 2014;19(7):20708. 10.2807/1560-7917.ES2014.19.7.20708 24576473

[4] Werber D Michaelis K Hausner M Sissolak D Wenzel J Bitzegeio J Ongoing outbreaks of hepatitis A among men who have sex with men (MSM), Berlin, November 2016 to January 2017 - linked to other German cities and European countries. Euro Surveill. 2017;22(5):30457. 10.2807/1560-7917.ES.2017.22.5.30457 28183391 PMC5388120

[5] Freidl GS Sonder GJ Bovée LP Friesema IH van Rijckevorsel GG Ruijs WL Hepatitis A outbreak among men who have sex with men (MSM) predominantly linked with the EuroPride, the Netherlands, July 2016 to February 2017. Euro Surveill. 2017;22(8):30468. 10.2807/1560-7917.ES.2017.22.8.30468 28251892 PMC5356436

[6] Manor Y Lewis M Ram D Daudi N Mor O Savion M Evidence for Hepatitis A virus endemic circulation in Israel despite universal toddlers’ vaccination since 1999 and low clinical incidence in all age groups. J Infect Dis. 2016;215(4):574-80. Available from: https://doi.org/10.1093/infdis/jiw611 28013247 10.1093/infdis/jiw611

[7] National Institute of Public Health and the Environment (RIVM) Hepatitis A Lab Network. (HAVNET). Protocol: Molecular detection and typing of VP1region of Hepatitis A vius (HAV). RIVM.[Accessed Jun 2017]. Available from: http://www.rivm.nl/en/Topics/H/HAVNET/Protocols/Typing_protocol_HAVNET_VP1P2A.org.

[8] Tamura K Stecher G Peterson D Filipski A Kumar S MEGA6: Molecular Evolutionary Genetics Analysis version 6.0. Mol Biol Evol. 2013;30(12):2725-9. 10.1093/molbev/mst197 24132122 PMC3840312

[9] Lavanchy D Viral hepatitis: global goals for vaccination. J Clin Virol. 2012;55(4):296-302. 10.1016/j.jcv.2012.08.022 22999800

[10] Levine H Kopel E Anis E Givon-Lavi N Dagan R The impact of a national routine immunisation programme initiated in 1999 on Hepatitis A incidence in Israel, 1993 to 2012. Euro Surveill. 2015;20(7). 10.2807/1560-7917.ES2015.20.7.21040 25719962

[11] Israel MoH. Refreshing the Ministry of Health guidelines concerning immunization against Viral Hepatitis (Hepatitis A). [Accessed June 18, 2017]; Available from: http://www.health.gov.il/English/News_and_Events/Spokespersons_Messages/Pages/07022013_1.aspx.

[12] World Health Organization (WHO). Hepatitis A outbreaks mostly affecting men who have sex with men – European Region and the Americas. WHO. Jun 2017. Available from: http://www.who.int/csr/don/07-june-2017-hepatitis-a/en/.

